# A multi‐year dormancy strategy in a cabbage beetle population in southeastern China

**DOI:** 10.1002/ece3.8900

**Published:** 2022-05-07

**Authors:** Jian‐Jun Tang, Xing‐Ping Liu, Hai‐Min He, Li‐Li Huang, Shao‐Hui Wu, Fang‐Sen Xue

**Affiliations:** ^1^ College of Computer and Information Engineering Jiangxi Agricultural University Nanchang China; ^2^ College of Forest Jiangxi Agricultural University Nanchang China; ^3^ Institute of Entomology Jiangxi Agricultural University Nanchang China; ^4^ Department of Ecology and Environment YuZhang Normal University Nanchang China; ^5^ Department of Entomology University of Georgia Tifton Georgia USA

**Keywords:** *Colaphellus bowringi*, photoperiod, prolonged diapause, temperature

## Abstract

The life cycle of the cabbage beetle *Colaphellus bowringi* in southeastern China is complex due to four options for adult development: summer diapause, winter diapause, prolonged diapsuse, and nondiapause. However, detailed information on the multi‐year emergence patterns of diapausing individuals in this beetle has not been documented. In this study, we monitored the adult emergence patterns of diapausing individuals and estimated the influence of the diapause‐inducing temperature and photoperiod on the incidence of prolonged diapause under seminatural conditions for several years. The duration of diapause for adults collected from the vegetable fields in different years varied from several months to 5 years. Approximately 25.9%–29.2% of individuals showed prolonged diapause (emergence more than 1 year after entering diapause) over the 5 years of observation. Furthermore, regardless of insect age, the emergence of diapausing adults from the soil always occurred between mid‐February and March in spring and between late August and mid‐October in autumn, when the host plants were available. The influence of diapause‐inducing temperatures (22, 25, and 28°C) combined with different photoperiods (L:D 12:12 h and L:D 14:10 h) on diapause duration was tested under seminatural conditions. Pairwise comparisons of diapause duration performed by the log‐rank test revealed that the low temperature of 22°C combined with the long photoperiod of L:D 14:10 h induced the longest diapause duration, whereas the low temperature of 22°C combined with the short photoperiod of L:D 12:12 h induced the highest proportion of prolonged diapause. This study indicates that *C*. *bowringi* adopts a multi‐year dormancy strategy to survive local environmental conditions and unpredictable risks.

## Graphical abstract

Our results showed that duration of diapause for adults collected in different years varied from several months to 5 years. Approximately 25.9%–29.2% of individuals showed prolonged diapause over the 5 years of observation. The temperature and photoperiod during induction have a significant influence on the incidence of prolonged diapause.

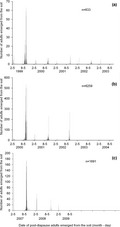

## 1 INTRODUCTION

Most insect species are subject to unpredictable habitat fluctuation, making it challenging for individuals to correctly time important life‐history events such as emergence and reproduction (Danks, 1987; Masaki, 1980; Tauber et al., 1986). One way to manage uncertainty is to spread the emergence of offspring across multiple years via prolonged diapause. Such a reproductive strategy avoids a situation in which individuals “put all their eggs into one basket”; this approach also potentially allows organisms to cope with unpredictable conditions (Hanski, 1988; Hopper, 1999; Matsuo, 2006; Waldbauer, 1978; Wise, 1980; Xue & Kallenborn, 1993). Understanding how organisms cope with and adapt to changes in their environment is a central theme in evolutionary ecology and is also of fundamental importance for assessing evolutionary and ecological dynamics (Parmesan, 2006).

Diapause duration (or intensity) is determined both by adaptive responses to local environmental conditions (such as the temperature, photoperiod, moisture, and host plant) that provide cues for terminating diapause and by plastic responses to the unpredictable temporal and spatial heterogeneity of habitats that drive a certain proportion of a population to extend their dormancy by entering prolonged diapause (Danks, 1987; Tauber et al., 1986).

The influence of diapause‐inducing temperatures and photoperiods experienced in the prediapause growth stages on the duration of diapause has been reported in a number of different insect species that undergo simple diapause (emergence in the next season or next year), which include the green lacewing *Chrysopa ocullata* (Nechols et al., 1987), the European corn borer, *Ostrinia nubilalis* (Beck, 1989), the fruit flies, *Drosophila auraria* (Kimura, 1990), the bruchid *Bruchidius atrolineatus* (Glitho et al., 1991), the Hessian fly *Mayetiola destructor* (Wellso, 1991), the Mediterranean tiger moth *Cymbalophora pudica* (Koštál & Hodek, 1997), the grape berry moth *Lobesia botrana* (Roditakis & Karandinos, 2001), the bug *Pyrrhocoris apterus* (Kalushkov et al., 2001), the corn stalk borer *Sesamia nonagrioides* (Fantinou et al., 2003), the yellow‐spotted longicorn beetle *Psacothea hilaris* (Asano et al., 2004), the cabbage butterfly *Pieris melete* (Xiao et al., 2006, 2008), the zygaenid moth *Pesuodopidorus fasciata* (Wu et al., 2006), the swallowtail *Sericinus montelus* (Wang et al., 2009), the cotton bollworm *Helicoverpa armigera* (Chen et al., 2014), and the Asian corn borer, *Ostrinia furnacalis* (Yang et al., 2014). However, the influence of diapause‐inducing temperature and photoperiod on the incidence of prolonged diapause has been reported only in a few insect species (Danks, 1987; Sullivan & Wallace, 1967).

The cabbage beetle, *Colaphellus bowringi* Baly (Coleoptera: Chrysomelidae), is an economically important defoliator of crucifers and is widely distributed in China (He et al., 2021; Tang et al., 2017; Zhang & Zhao, 1996). In the present study, the experimental population was collected from the mountainous areas of Jiangxi Province, Southeast China. This population undergoes aestivating and hibernating imaginal diapause in the soil and exhibits a short‐day response (i.e., develops in response to a short daylength) (Xue, Li, et al., 2002). All adults entered diapause at temperatures ≤20°C regardless of the photoperiod. The diapause‐averting influence of short daylengths was expressed only at temperatures above 20°C, and high temperatures strongly weakened the diapause‐inducing effects of long daylengths (He et al., 2018; Xue et al., 2002). In the field, this beetle showed two distinct infestation peaks in this region: one occurring in spring, between March and April. In spring, the mean daily temperature in April was below 20°C in the region. Thus most individuals of the spring generation entered summer diapause mainly in response to low temperature. A very small proportion of individuals could develop and produce the second generation. In autumn, the early‐hatching larvae occurred at the end of August when they were subjected to high temperatures (above 25°C). Most of these individuals developed without diapause and produced the second‐generation larvae in mid‐September. These larvae still underwent temperatures above 20°C and some adults continued to reproduce. The early‐hatching individuals of the third generation occurred in mid‐October and entered diapause when temperatures were around 20°C (Xue, Li, et al., 2002). In all our experiments on diapause induction of *C*. *bowringi*, total emergence was never achieved; some individuals always entered diapause irrespective of the rearing conditions, suggesting that the onset of diapause in these individuals was independent of the environment (Xue, Li, et al., 2002; Xue, Spieth, et al., 2002). Furthermore, the adults that entered summer and winter diapause at the same time or in the same season showed a great difference between individuals in diapause duration (from several months to more than 2 years) (Wang et al., 2006; Wei et al., 2010; Xue & Kallenborn, 1993; Xue, Spieth, et al., 2002; Yang et al., 2007). However, detailed information on the multi‐year emergence patterns of diapausing individuals in this beetle has not been documented. The objective of this study was to monitor the adult emergence patterns of diapausing individuals (in terms of diapause duration) and to estimate the influence of the diapause‐inducing temperature and photoperiod on the incidence of prolonged diapause under seminatural conditions.

## 2 MATERIALS AND METHODS

### 2.1 Adult collection

Adults of *C*. *bowringi* adults were collected from the leaves of vegetable fields in late November 1998, 1999, and 2006 in Xiu‐Shui County, Jiangxi Province, PR China (29°1′N, 114°4′E), and those collected adults were destined for diapause. Adults were then transferred to large glass bottles (diameter: 18 cm; height: 32 cm) containing moist soil to burrow into during dormancy after several days of feeding. Each glass bottle contained at least 100 diapausing individuals. The glass bottles were maintained under seminatural conditions (a covered, open‐air room with natural temperature and photoperiod fluctuations) at Jiangxi Agricultural University, Nanchang, Jiangxi Province (28°46’ N, 115°59’ E).

### 2.2 Observation of diapause termination under seminatural conditions

We recorded the adult emergence patterns of naturally diapausing individuals collected in 1998 and 1999 for 5 consecutive years and those of diapausing individuals collected in 2006 for 3 consecutive years. When the postdiapause adults emerged from the soil in spring and autumn, we recorded their emergence time twice daily at 12‐h intervals. A total of 633 diapausing adults collected in 1998 emerged from the soil from 1999 to 2003, those diapausing adults were placed in seven glass bottles with 65–111 individuals each glass bottle. A total of 6259 diapausing adults collected in 1999 emerged from the soil from 2000 to 2004, those diapausing adults were placed in 46 glass bottles with 97–178 individuals each glass bottle. A total of 1691 diapausing adults collected in 2006 emerged from the soil from 2007 to 2009, those diapausing adults were placed in 13 glass bottles with 69–166 individuals each glass bottle.

### 2.3 Influence of diapause‐inducing temperature and photoperiod on the duration of diapause

To estimate the effect of diapause‐inducing temperature and photoperiod on diapause duration, newly hatched larvae produced by postdiapause adults that emerged from the soil in mid‐October 2006 were reared in plastic rearing boxes (17.5×12.5×6.5 cm) with mature leaves of radish (*Raphanus sativus* var. *longipinnatus*) under an intermediate photoperiod of L:D 12:12 (12 h light:12 h dark) and a long photoperiod of L:D 14:10 at 22, 25, and 28°C until the adults entered diapause. Each box contained at least 60 individuals. Each treatment was replicated three to ten times. This experiment was performed in illuminated incubators (LRH‐250‐GS, Guangdong Medical Appliances Plant). The light intensity during the photophase was approximately 1.97 W m**^﹣2^**, and the variation in temperature was ±1°C. The diapausing adults obtained were transferred to large glass bottles containing soil to burrow into during dormancy. The bottles were maintained under seminatural conditions at Jiangxi Agricultural University, Nanchang, Jiangxi Province (28°46’ N, 115°59’ E). Then, we recorded the adult emergence patterns for 3 consecutive years when diapausing individuals began to emerge from the soil.

Climatic data (the mean daily temperatures (°C) experienced by the diapausing adults) are shown in Table 1. The climatic data were collected from the Weather Station of Jiangxi Agricultural University (28°46’ N, 115°59’ E).

**TABLE 1:** Climatic data (mean daily temperature (°C)) collected from the Weather Station of Jiangxi Agricultural University where the experiments with *Colaphellus bowringi* were performed

	Jan	Feb	Mar	Apr	May	Jun	Jul	Aug	Sep	Oct	Nov	Dec
1998	4.4	9.4	10.5	21.1	23.4	24.9	29.3	30.1	25.2	21.4	16.3	9.6
1999	7.4	10.2	10.6	18	22.1	25.7	27.4	27.1	26.5	20.2	13.7	8.7
2000	5.1	6	12.7	17.6	24.1	25.8	30.1	28.3	25	19.3	11.9	8.9
2001	6.7	8.4	12.7	17.1	23.3	25.6	30.6	27.1	25.8	20.9	13.8	6.6
2002	8.8	10.2	13.9	18.1	21.1	27.2	29	27.8	24.4	19.4	14	7.4
2003	6	8.9	11.4	17.9	22.7	26.1	31.6	30.2	26.7	19.8	14	7.9
2004	5.7	11.1	11.6	19.6	23.4	25.8	29.7	28.7	25.2	20.3	15.5	8.9
2005	3.7	4.7	10.7	20.5	22.8	27.5	30.4	28.5	27.1	19.9	15.6	7
2006	6.6	6.5	12.7	19.4	22.8	26	30	30	24.3	22.1	15	8.1
2007	5.7	12.1	13.7	17.5	25.2	26.5	30.9	29.6	24.7	21.1	14.3	9.2
2008	3.5	5.5	14.7	18.7	24.3	25.8	30.1	29.5	27	21.3	13.4	8.7
2009	5.1	10.8	12.1	18.7	23.5	27.8	29.7	29.4	26.9	22.5	11.2	7.3

### 2.4 Statistical analysis

Statistical analyses were conducted using the SPSS 22.0 statistical software package (IBM, http://www.ibm.com). To estimate the influence of different combinations of temperature and photoperiod on diapause duration, we adopted the Kaplan–Meier survival analysis. In this analysis, the diapause‐terminating function provides the probability of diapause as a function of time. The function gives the probability of the diapause termination time for diapausing individuals that emerged from the soil at 3 years. At the time provided by the function, more than 90% diapausing individuals would have emerged from the soil. In this analysis, the *p* values of pairwise comparisons were calculated using the log‐rank (Mantel–Cox) test.

## 3 RESULTS

### 3.1 Adult emergence patterns of diapausing individuals under seminatural conditions

**FIGURE 1:**
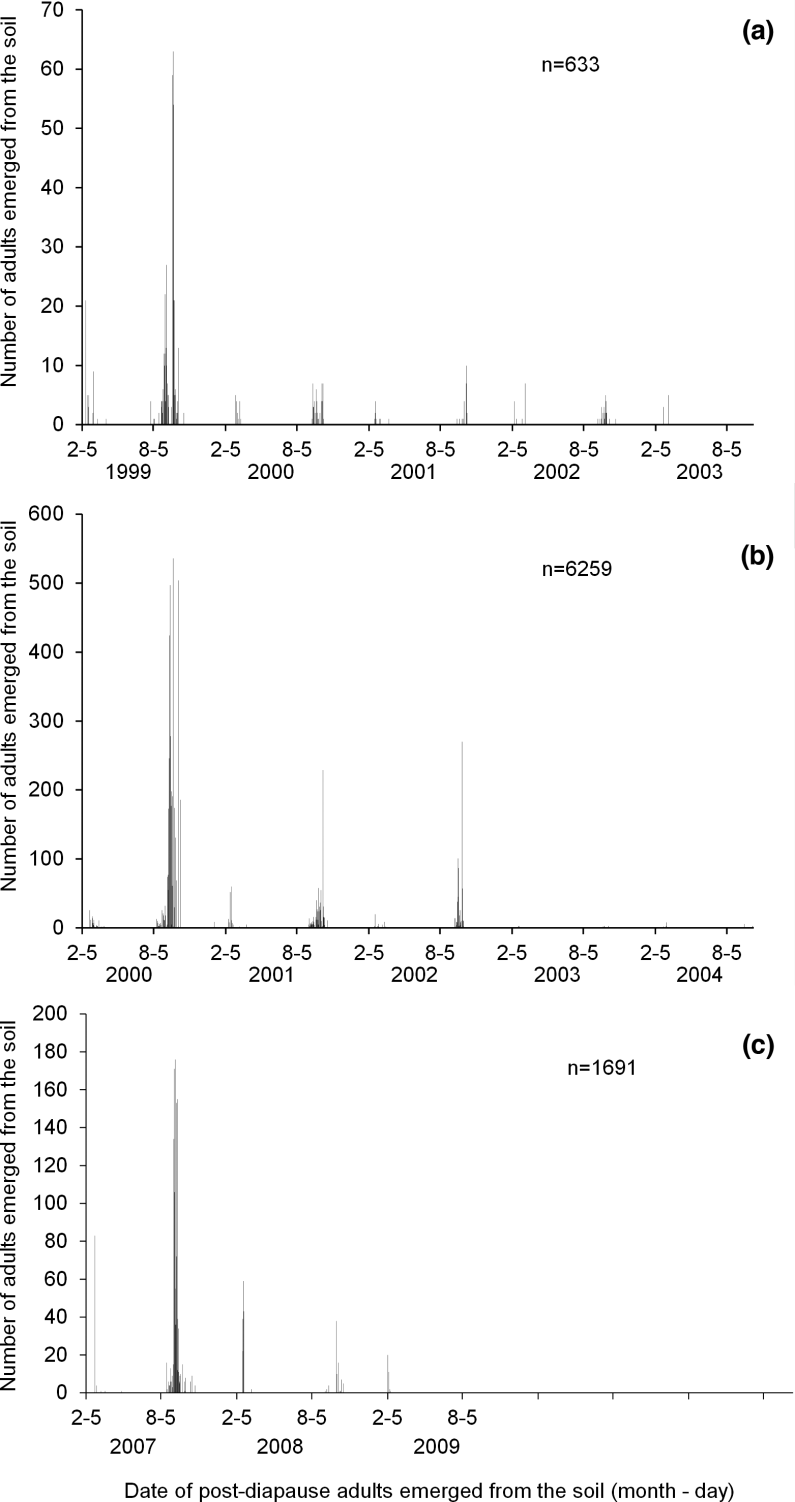
Emergence patterns of diapausing individuals of the cabbage beetle *Colaphellus bowringi* collected in vegetable gardens in late November 1998 (a), 1999 (b), and 2006 (c)

**FIGURE 2:**
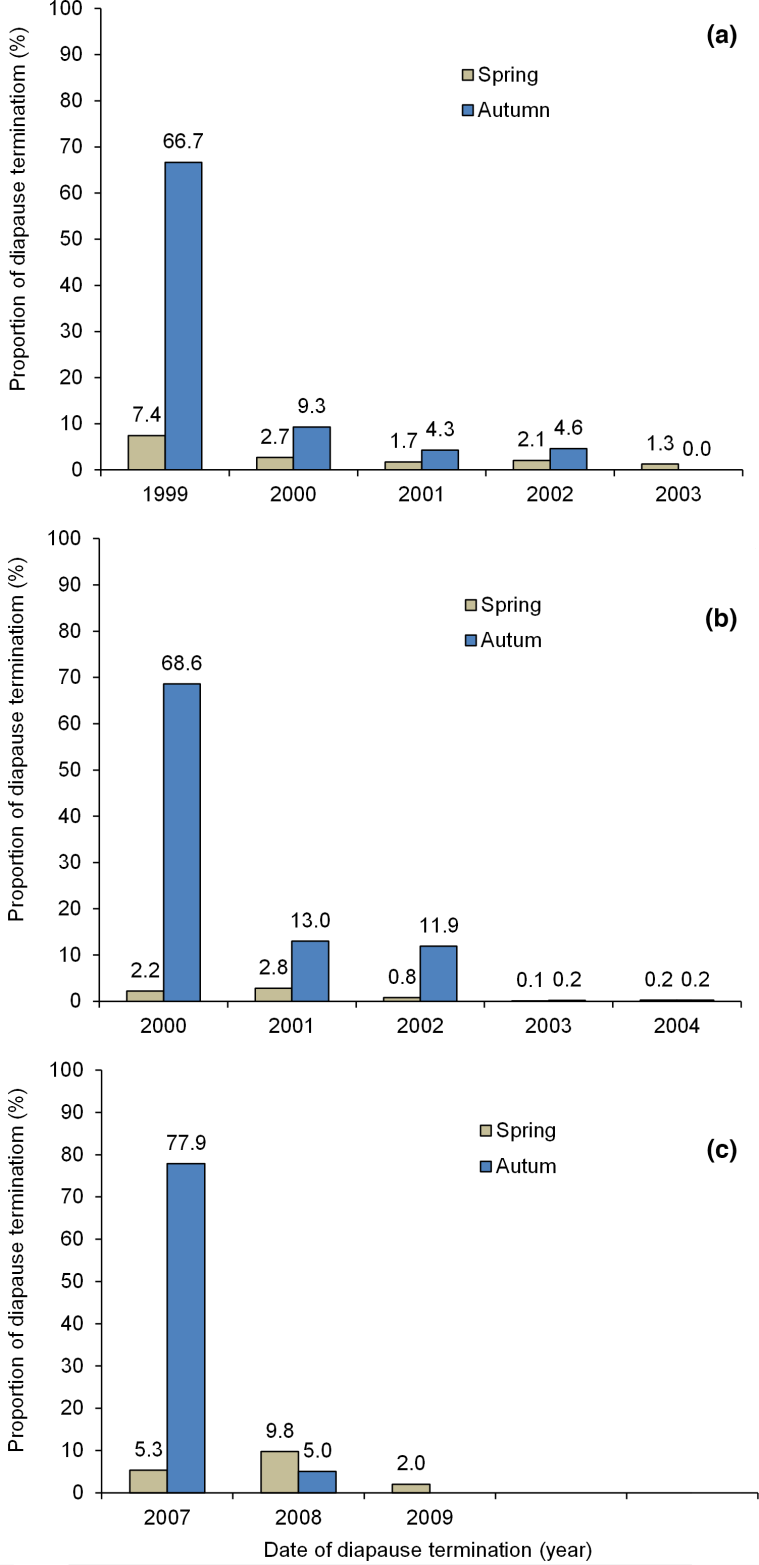
Termination of diapausing adults of the cabbage beetle *Colaphellus bowringi* collected in late November 1998 (a),1999 (b), and 2006 (c)

The adult emergence patterns of diapausing individuals collected in 1998 (Figure 1a) and 1999 (Figure 1b) showed vast variation in diapause duration, from 76 to 1560 days in 1998 (Figure 1a) and from 68 to 1777 days in 1999 (Figure 1b). Furthermore, regardless of the age of the insects, adult emergence always occurred between early February and late March in spring and between late August and mid‐October in autumn. Among a total of 663 diapausing individuals that entered winter diapause in late November 1998, 7.4% emerged from soil in spring, and 66.7% emerged in autumn in 1999; 2.7% emerged in spring, and 9.3% emerged in autumn in 2000; 1.7% emerged in spring, and 4.3% emerged in autumn in 2001; 2.1% emerged in spring, and 4.6% emerged in autumn in 2002; and 1.3% emerged in spring in 2003 (Figure 2a). In this group, diapause lasted for up to 5 years, and 25.9% of individuals showed prolonged diapause (emergence after more than 1 year). Among a total of 6295 individuals that entered winter diapause in late November 1999, 2.2% emerged from soil in spring, and 68.6% emerged in autumn in 2000; 2.8% emerged in spring, and 13% emerged in autumn in 2001; 0.8% emerged in spring, and 11.9% emerged in autumn in 2002; 0.1% emerged in spring, and 0.2% emerged in autumn in 2003; and 0.2% emerged in spring, and 0.2% emerged in autumn in 2004 (Figure 2b). In this group, similar to the group that entered diapause in 1998, diapause lasted for 5 years, and 29.2% of individuals showed prolonged diapause.

A total of 1691 diapausing individuals that entered winter diapause in late November 2006 also showed extended emergence, with diapause durations ranging from several months to 3 years (Figure 1c). Their emergence also occurred between mid‐February and March in spring and between late August and early October in autumn. Approximately 5.3% of diapausing individuals emerged from the soil in spring, and 77.9% emerged in autumn in 2007; 9.8% emerged in spring, and 5% emerged in autumn in 2008; and 2% emerged in spring in 2009 (Figure 2c). In this group, 16.8% of individuals showed prolonged diapause.

### 3.2 Influence of diapause‐inducing temperature and photoperiod on the duration of diapause

The duration of diapause induced by different temperature and photoperiod combinations varied (Table 2). The low temperature of 22°C combined with the long photoperiod of L:D 14:10 induced the longest diapause duration (274.2 ± 5.1 days), whereas the high temperature of 28°C combined with the short photoperiod of L:D 12:12 induced the shortest diapause duration (154.2 ± 6.8 days). The low temperature of 22°C combined with the short photoperiod of L:D 12:12 induced the highest proportion of prolonged diapause (15.7%), whereas the high temperature of 28°C combined with the short photoperiod of L:D 12:12 induced the lowest proportion of prolonged diapause (1.4%).

**TABLE 2:** The duration of diapause in *Colaphellus bowringi* when diapause was induced by different temperature and photoperiod combinations under seminatural conditions in the laboratory

Diapause‐inducing temperature (°C)	Diapause‐inducing photoperiod	N^a^	Duration of diapause (days)			Prolonged diapause^b^ %
			Range	Median	Mean ± SE	
22	L:D14:10	442	86–806	283	274.2 ± 5.1	12.1
	L:D12:12	529	86–798	283	263.1 ± 5.4	15.7
25	L:D14:10	606	90–681	142	209.2 ± 4.4	8.8
	L:D12:12	122	90–455	114	168.9 ± 10.2	10.4
28	L:D14:10	382	94–686	133	202.5 ± 6.0	12.2
	L:D12:12	169	94–459	120	154.2 ± 6.8	1.4

^a^ Number of diapausing adults surviving to emerge from the soil.

^b^ Percentage of diapausing adults that emerged from the soil more than 1 year after diapause.

**FIGURE 3:**
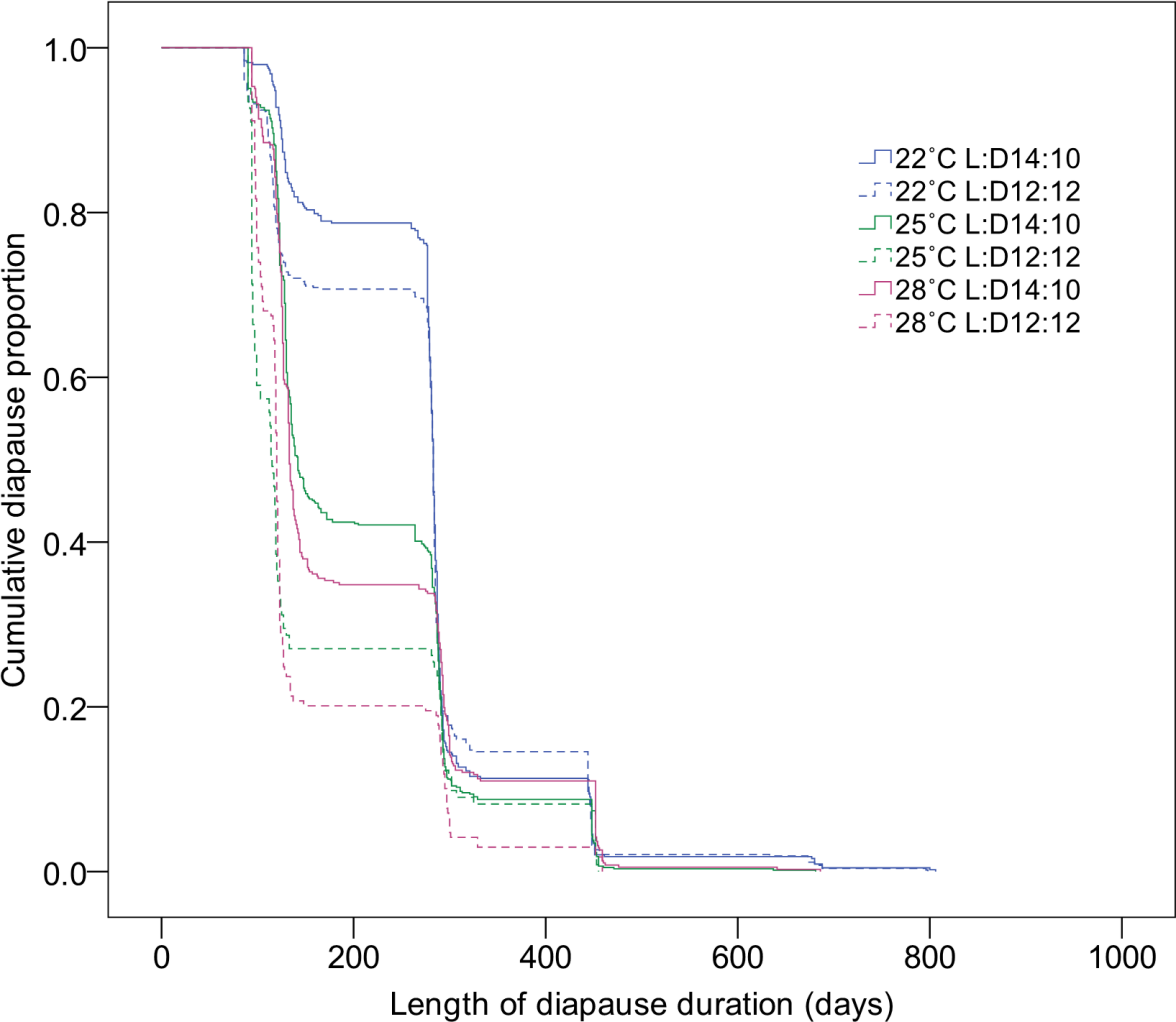
Diapause–termination curves of the cabbage beetle *Colaphellus bowringi* under different combinations of temperature and photoperiod performed by Kaplan–Meier survival analysis

The diapause‐terminating curves of *C*. *bowringi* obtained by Kaplan–Meier survival analysis also showed that diapause termination occurred the earliest when the high temperature of 28°C was combined with the short photoperiod of L:D 12:12 during induction and the slowest at a temperature of 22°C combined with the long photoperiod of L:D 14:10 (Figure 3). Pairwise comparisons of diapause duration among different combinations of temperature and photoperiod performed by the log‐rank (Mantel–Cox) method revealed that there were significant differences in diapause duration (*p* < .05) except for the treatments between L:D12:12 and L:D14:10 at 22°C, between 28°C, L:D14:10 and 22C, L:D12:12, and between 25°C and 28°C at L:D14:10 (*p* > .05) (Table 3).

**TABLE 3:** Pairwise comparisons of diapause duration in *Colaphellus bowringi* performed by the log rank (Mantel–Cox) test, in which diapause was induced by different combinations of temperature and photoperiod

	22°C L:D14:10		22°C L:D12:12		25°C L:D14:10		25°C L:D12:12		28°C L:D14:10	
	Chi‐square	*p*	Chi‐square	*p*	Chi‐square	*p*	Chi‐square	*p*	Chi‐square	*p*
22°C L:D12:12	0.877	.**349**								
25°C L:D14:10	18.395	.000	8.853	.003						
25°C L:D12:12	24.047	.000	19.135	.000	14.402	.000				
28°C L:D14:10	7.276	.007	2.762	.**097**	1.459	.**227**	24.597	.000		
28°C L:D12:12	75.622	.000	55.135	.000	39.752	.000	0.243	.622	44.195	.000

Note

The bold values mean no significant differences.

## 4 DISCUSSION

Prolonged diapause in insects is a common phenomenon and is regarded as a survival strategy (Danks, 1987; Menu & Debouzie, 1993; Moraiti et al., 2014; Saulich, 2010). Danks (1987) reported 150 insect species showing prolonged diapause, and the duration of the prolonged diapause generally varied from 2 to 4 years. However, only a few species with prolonged diapause have been studied for several years. In the chestnut weevil *Curculio elephas*, adult emergence spread over 3 or 4 years due to prolonged larval diapause in some individuals (Menu, 1993, see Table 1 therein). In Colorado potato beetles, *Leptinotarsa decemlineata*, most (≈70%) beetles that underwent prolonged dormancy emerged after two winters in diapause; the majority of the remainder (≈29%) emerged after 3–7 years in diapause; and one beetle emerged after 9 years (Tauber & Tauber, 2002). In the seed‐predatory weevil *Exechesops leucopis*, the duration of larval diapause varied from 1 to 4 years; 52.5% of individuals emerged in the first year, 44% in the second year, 3% in the third year, and 0.5% in the fourth year (Matsuo, 2006). In the present study, our investigations showed the occurrence of prolonged diapause for up to 5 years in a population of *C*. *bowringi* from Southeast China. The multi‐year emergence strategy of diapausing adults in the southeastern population may serve a survival strategy in response to unpredictable conditions. In the field, in addition to climatic changes, the greatest threat that the beetle faces is the food source availability, as farmers may opt to growth the crucifer plants or non‐host crops depending on market values and farming conditions, and they may thin out the seedlings and harvest the crops at regular or irregular intervals. Furthermore, farmers may apply insecticide to prevent the damage from pests. These actions may greatly affect the insect populations in different seasons and years. Therefore, the multi‐year emergence pattern of diapausing adults potentially allows the cabbage beetle to cope with unpredictable conditions and to avoid the catastrophic elimination of an entire population.

We found that the emergence of diapausing adults from the soil always occurred between mid‐February and March in spring and between late August and mid‐October in autumn. In the local subtropical climate, cruciferous crops, which are the main food sources for *C*. *bowringi*, are generally grown in spring and autumn. This emergence pattern thus ensures that the species synchronizes its development and reproduction with the growth seasons of the cruciferous crops. Our study showed that the emergence pattern of diapausing individuals collected in 1998, 1999, and 2006 were similar with a peak occurring in autumn of the following year (Figure 1). This suggests that the emergence pattern of diapausing individuals in *C*. *bowringi* is probably genetically determined which needs to be verified in future studies.

Previously, Sullivan and Wallace (1967) reported the interaction of temperature and photoperiod in the induction of prolonged diapause in the European pine sawfly, *Neodiprion sertifer*. When cocoons (diapausing prepupae) from short‐day rearings were exposed to 10°C instead of 2l°C, a small proportion of the prepupal larvae underwent the normal or short diapause, but the majority entered an intense or prolonged type of diapause, suggesting that prolonged diapause was caused by short daylength and low temperature. In the present study, the relatively low temperature of 22°C combined with the long photoperiod of L:D 14:10 induced the longest diapause duration, whereas the low temperature of 22°C combined with the short photoperiod of L:D 12:12 induced the highest proportion of prolonged diapause. Our study further expanded the study of Sullivan and Wallace (1967) study by looking at the influence of different combinations of temperature and photoperiod on prolonged diapause.

For most insects with an extensive geographical distribution, the incidence of prolonged diapause is variable among populations and is related to the predictability, frequency and severity of unfavorable periods (Danks, 1987; Tauber & Tauber, 2002). In the Colorado potato beetle *L*. *decemlineata*, the incidence of prolonged diapause in the population from the western United States was 22%, whereas the incidence of prolonged diapause in the population from the northeastern United States was 2% (Tauber & Tauber, 2002). This discrepancy occurs because beetles in the western United States commonly occur on wild, solanaceous host plants in their drought‐prone native habitat, whereas beetles in the northeastern United States are not commonly found on wild, solanaceous plants (Tauber & Tauber, 2002). In the present study, the southeastern population of *C*. *bowringi* showed a multi‐year emergence pattern due to prolonged diapause in some individuals (emergence spread over 5 years). Interestingly, the naturally diapausing adults of *C*. *bowringi* collected in Harbin city (45°8’ N, 126°6’ E), northern China, showed simple diapause, and all emerged from the soil between April and early May (Hu et al., 2008). The incidence of diapause in the northern HB population was determined by temperature and was independent of photoperiod; almost all individuals entered diapause at 28°C (Lai et al., 2008), thus showing a life history with one generation per year. According to field observations, beetles in the southeastern region commonly occur on cultured vegetable crops (such as radish, *Raphanus sativus* var. *longipinnatus*, and cabbage, *Brassica chinensis*), whereas beetles in the northern region commonly occur on wild host plants (such as garden cress, *Lepidium apetalum*, and India yellow cress, *Rorippa indica*) (Tang et al., 2017). Furthermore, cultured vegetable crops are generally planted in spring and autumn in the southeastern region because the high‐temperature conditions during summer are not suitable for their growth, whereas wild host plants in the northern region commonly grow between April and June. Moreover, winter in Harbin city is extremely cold, and the daily mean minimum temperature in the coldest January is below minus 20°C. However, winter in Nanchang city is mild, with a mean daily temperature between 3.5 and 8.8°C in the coldest month, January (Table 1). Therefore, these differences may suggest that the prolonged diapause in *C*. *bowringi* is due to local climatic variability.

In some insect species, diapause is longer in larger individuals than in small individuals, as seen in the spruce cone moth *Laspeyresia strobilella* (Bakke, 1971), the yucca moth *Prodoxus y*‐*inversus* (Powell, 1989), the desert bee *Perdita portalis* (Danforth, 1999), the chestnut weevil *C*. *elephas* (Menu & Desouhant, 2002), the seed‐predatory weevil *E*. *leucopis* (Matsuo, 2006), and the cabbage beetle *C*. *bowringi* (Wei et al., 2010). Unfortunately, the adults of *C*. *bowringi* that emerged from soil were not weighed in the present study because we did not realize the importance of this factor when conducting the experiments. However, we investigated the correlation between the diapause duration and body weight of postdiapause adults in a *C*. *bowringi* population collected in Taian city (36° 2′N, 117° 1′E). We found that the mean body weight of postdiapause adults with a long diapause duration of 21 months in the Taian population was significantly greater than that of postdiapause adults with a shorter diapause duration of 5, 11, or 17 months (Wei et al., 2010). Size dependency in the length of diapause duration has been explained mainly by the amount of energy resources that individuals can metabolize during diapause development, because prolonged diapause is more costly in terms of relative weight loss for smaller individuals (Matsuo, 2006).

It is generally difficult to run experiments for years to test the costs of prolonged diapause. However, our previous experiment in this southeast population of *C*. *bowringi* revealed that the mean total egg production per female with a long diapause period of 16, 22, 29, or 34 months was significantly higher than that per female with a short diapause period of 5 months and that there were no significant differences in the mean total egg production per female among diapausing adults with a diapause duration of 16, 22, 29, or 34 months (Wang et al., 2006). These results suggest that prolonged diapause has a positive influence on fecundity. Further studies will be needed to confirm the effect of prolonged diapause on reproductive development and evaluate its influence on offspring fitness.

## 5 CONCLUSSION

Our study showed that the emergence of diapausing adults of *C*. *bowringi* in southeastern China spread over 5 years due to prolonged diapause in some individuals, suggesting a survival strategy in response to unpredictable conditions. The emergence of diapausing adults from the soil always occurred in spring and autumn, when the host plants were available. The study also revealed that the temperature and photoperiod during induction had a significant influence on the incidence of prolonged diapause. A better understanding of the multi‐year dormancy strategy of *C*. *bowringi* may provide valuable information in predicting the population dynamics of the beetle predation on vegetable crops for developing a suitable and practical management strategy. The variable multi‐year emergence patterns as prolonged diapause in this insect provides a good example in studying insect evolutionary ecology as a survival strategy in response to unpredictable conditions.

## CONFLICT OF INTEREST

The authors declare that they have no conflict of interest.

## AUTHOR CONTRIBUTION

**Jianjun Tang:** Data curation (equal). **Xingping Liu:** Investigation (equal). **Hai‐Min He:** Investigation (equal). **Li‐Li Huang:** Investigation (equal). **Shao‐Hui Wu:** Writing – original draft (equal). **Fangsen Xue:** Conceptualization (equal); Writing – original draft (equal).

## Data Availability

Empirical data have been archived in DataDryad: https://doi.org/10.5061/dryad.c866t1g8x.
